# Effects of flavonoids on expression of genes involved in cell cycle regulation and DNA replication in human fibroblasts

**DOI:** 10.1007/s11010-015-2458-3

**Published:** 2015-05-24

**Authors:** Marta Moskot, Joanna Jakóbkiewicz-Banecka, Elwira Smolińska, Ewa Piotrowska, Grzegorz Węgrzyn, Magdalena Gabig-Cimińska

**Affiliations:** 1 Laboratory of Molecular Biology (affiliated with the University of Gdańsk), Institute of Biochemistry and Biophysics, Polish Academy of Sciences, Wita Stwosza 59, 80-308 Gdańsk, Poland; 2 Department of Molecular Biology, University of Gdańsk, Wita Stwosza 59, 80-308 Gdańsk, Poland

**Keywords:** Flavonoids, Cell cycle regulation, DNA replication process, Gene expression profiling, Cell growth

## Abstract

Flavonoids have been studied as potential agents in medicine for many years. Among them, genistein was found to be active in various biological systems, mainly in prevention of cancer. Our recent work supported the idea that genistein also impacts multiple cellular processes in healthy fibroblasts; however, its effects on cell cycle-related pathways remained to be elucidated. Thus, in this work, high throughput screening with microarrays coupled to real-time quantitative Reverse Transcription PCR analyses was employed to study the changes in expression of key genes associated with cell cycle regulation and/or DNA replication in response to genistein, kaempferol, daidzein, and mixtures of genistein and either kaempferol or daidzein. Among them, genistein was found as the most significantly modulating, in a time- and dose-dependent manner, compound of activity of studied genes, whose products are involved in different phases of the cell cycle and/or in regulatory processes important for DNA replication and cell growth. It considerably reduced the efficiency of expression of genes coding for MCM2-7 and MCM10 helicases, as well as some other proteins involved in the S phase control. In addition, genistein caused cell cycle arrest in the G2/M phase, which was accompanied by activation of *CDKN1A*, *CDKN1C*, *CDKN2A*, *CDKN2B*, *CDKN2C,* and *GADD45A* genes, as well as down-regulation of several mRNAs specific for this stage, demonstrated by transcriptomic assessments. We believe that studies described in this paper will be helpful in elucidating molecular mechanisms of action of genistein as modulator of cell cycle and inhibitor of DNA replication in humans.

## Introduction

Compounds produced by one kind of organism (one species) can significantly influence physiology of other species. Interesting examples are plant-derived metabolites, which have various biological activities and can affect basic biological processes in humans [1]. In fact, such compounds can be taken by humans with food, which in turn has an impact on health condition of the human body. Examples of bioactive plant metabolites are flavonoids [2]. This group consists of compounds that are considered as therapeutic molecules for cancer, infections, and some genetic diseases, for example, cystic fibrosis and mucopolysaccharidoses [3–5]. Therefore, determining mechanisms of their biological activities is very important to assess safety of these compounds and to develop optimal therapeutic procedures. Interestingly, flavonoids can influence cell cycle and DNA metabolism, but detailed mechanisms of their actions are not completely understood [2]. Effects of plant metabolites, among them genistein, on cell cycle and DNA replication have been studied in the light of human health, especially prevention of cancer [6–11]. Genistein was found to inhibit cell division cycle and DNA replication in eukaryotic cells when used at sufficiently high concentrations [12, 13]. It was suggested that such an action might be caused by blocking activity of topoisomerase [14–17]. Genistein was also shown to exhibit biological activity through inhibition of various kinases [14, 18].

Recently, in order to investigate more extensively the effects of genistein in cell culture models, we have used DNA microarrays to assess the global effects of this compound on RNA expression in fibroblasts obtained from a healthy adult donor [19]. These studies supported the idea that genistein impacts multiple cellular pathways; however, its specificity and effects on cell cycle-related pathways remained to be delineated. The cell cycle or cell division cycle is the series of events that takes place in a cell leading to its division and duplication. It is controlled by numerous mechanisms ensuring correct cell splitting. Elucidation of the global pattern of molecular changes caused by genistein in respect to cell proliferation and DNA replication is desired in the light of the complex nature of genistein’s biological activity as well as its unique effects in various biological systems, above all in humans. We found this topic interesting in respect not only to genistein, but also to other flavonoids, kaempferol, and daidzein, likewise mixtures of genistein and either of them. We employed human cell line of fibroblasts, which should open new possibilities to assess cell cycle regulatory processes operating in normal, untransformed, cells. The use of a cell culture model that exhibits a finite proliferative capacity has the advantage of providing a controlled environment to study a wide variety of cellular phenomena. It has also the inherent limitation of isolating cells from the regulatory elements that might be provided by other types of cells. Among different cells types (such as glial cells, keratinocytes, vascular smooth muscle cells, lens cells, endothelial cells, lymphocytes, liver, and melanocytes), fibroblasts from normal individuals exhibit most limited replicative life-span in the culture and therefore remain a powerful tool for a variety of studies.

We assumed that such studies have to contribute to both extending our basic knowledge on the influence of flavonoids on human cell cycle and identifying potential adverse effects on these compounds (used in various therapies) in normal cells. To address these issues, we utilized transcriptomic approach, i.e., a microarray gene profiling and a real-time quantitative Reverse Transcription PCR (real-time qRT-PCR) to examine gene expression, as well as cell viability and cell cycle progression tests to study development of fibroblasts exposed to different concentrations of tested compounds.

## Materials and methods

### Cell lines, culture media, supplements, and flavonoid solutions

Human Dermal Fibroblasts, adult (HDFa) (Cascade Biologics, Portland, USA) were cultured in Dulbecco’s modified Eagle’s medium (DMEM, Sigma-Aldrich, Steinheim, Germany) supplemented with 10 % fetal bovine serum (FBS) and 1 % antibiotic/antimycotic solution (Sigma-Aldrich, Steinheim, Germany), at 37 °C and 5 % CO_2_. Genistein was synthesized at the Pharmaceutical Research Institute (Warsaw, Poland), while kaempferol and daidzein were obtained from Sigma-Aldrich (Steinheim, Germany). Tested flavonoids were dissolved in dimethyl sulfoxide (DMSO) and added to cell cultures at the indicated final concentrations as determined in previous studies [20, 21]. For experimental procedures, cells were plated at approximately 80 % confluence. After overnight incubation, culture medium was replaced with fresh medium either flavonoid-free, containing DMSO at a final concentration of 0.05 % (control, K), or the one supplemented with appropriate amount of tested flavonoids. The experimental treatment was 24 and 48 h period.

### RNA extraction

Total RNA was extracted from cells using the High Pure RNA Isolation Kit (Roche Applied Science, Indianapolis, USA) following the manufacturer’s instructions. The quality and quantity of each RNA sample was evaluated using the RNA 6000 Nano Assay on the Agilent 2100 Bioanalyser (Agilent Technologies Inc., USA).

### Microarray assays for mRNA analysis

Whole genome microarray analysis of three biological replicates (*n*) was performed using Illumina’s Human HT-12v4 Expression BeadChips (Illumina Inc., USA). The assay performance, data extraction, and statistical analysis were performed as previously described [19]. Gene Ontology analysis and data visualization were performed using the web tools GOrilla (http://cbl-gorilla.cs.technion.ac.il/) and REViGO (http://revigo.irb.hr/) restricting the output to biological process and cell compartment. Gene set enrichment analysis (GSEA) was performed on the up-regulated and down-regulated gene lists, separately as previously described [22, 23]. A nominal *P* value <0.01 and a false discovery rate (FDR) <0.25 were used to assess the significance of the enrichment scores. The microarray expression data used in this study have GEO accession numbers GSE34074 and GSE43692.

### Real-time quantitative RT-PCR array for mRNA analysis

Total RNA was retrotranscribed with Transcriptor First-Strand cDNA Synthesis Kit (Roche Applied Science, Indianapolis, USA). Real-time qRT-PCR was carried out with Real-Time ready Custom Panel (cat no. 05532914001, config. no. 100064133; Roche Applied Science, Indianapolis, USA) and the LightCycler^®^ 480 Probes Master (Roche Applied Science, Indianapolis, USA) using the Light Cycler 480 II detection system (Roche). Expression values were normalized against three control genes *B2M, GAPDH,* and *RPLP0* of constant expression level. For both microarray and real-time qRT-PCR analyses, a fold change (FC) greater or equal to 1.3 and below 0.7 was considered as a relevant criterion for genes being significantly differentially expressed.

### Cytotoxicity and proliferation assay

MTT (3-[4,5-dimethylthiazol-2-yl]-2,5-diphenyltetrazolium bromide) assay was performed to estimate cell growth and proliferation. Cells were plated in flat-bottomed 96-well plates and treated with 30, 60, and 100 μM of genistein or 0.05 % DMSO as control for 24, 48, 72 h, and 7 days at 37 °C. After incubation period, medium was replaced with RPMI (Sigma-Aldrich) supplemented with MTT (1 mg/ml) for another 4 h. The purple formazan crystals were dissolved in 150 ml DMSO, and absorbance was determined at 570 nm using Wallac 1420 Multilabel Counter (Perkin Elmer).

### Cell viability assessment

10^5^ fibroblasts were seeded in triplicate in 6-well plates. The medium was changed the following day and supplemented with 30, 60, and 100 μM of genistein or 0.05 % DMSO as control for 24, 48, and 72 h. Cells were counted, and viability was estimated by MUSE^®^ Cell Analyzer (Merck Millipore, Germany) and Muse^®^ Count & Viability Assay Kit (Merck Millipore, Germany). An average of 2000 cells was analyzed for each condition.

### Fibroblast migration assay

To study the effect of genistein on cell migration, 5 × 10^4^ fibroblasts were seeded in triplicate in 6-well plates. The dishes were incubated at 37 °C until cells reach 100 % confluence to form a monolayer. A pipet tip was used to create a scratch of the cell monolayer, after that the plate was washed once and replaced with the desired medium, i.e., supplemented with 30, 60, and 100 μM of genistein or 0.05 % DMSO as control. The rate of cell migration was determined for the cells by measuring the distance traveled during the desired time frame of incubation, i.e., at 0, 6, 12, 18, and 24 h.

### Cell cycle assay

The effect of flavonoids on cell cycle was evaluated by seeding fibroblasts into 6-well plates at a density of 1 × 10^4^ cells per well. After 24 h, the medium was replaced with fresh one containing flavonoids. Cell cycle phase was determined by MUSE^®^ Cell Analyzer (Merck Millipore, Germany) using a Muse^®^ Cell Cycle Assay Kit (Merck Millipore, Germany) according to the manufacturer’s instructions. An average of 10,000 cells was analyzed for each condition. Triplicate independent experiments were conducted.

## Results

### Microarray expression analysis of cell cycle regulation and/or DNA replication-related genes

Whole genome microarray analysis of gene expression was performed in our prior experiments with human fibroblasts treated with genistein, daidzein, kaempferol, and their mixtures ([19]; GEO accession numbers GSE34074 and GSE43692). In this work, cell cycle and/or DNA replication-related genes (retrieved from: GO:0000278—mitotic cell cycle; KEGG:hsa04110—cell cycle; GO:0055133—DNA replication; KEGG:hsa03030—DNA replication) were filtered based on desired patterns of expression, up- and down-regulation at various experimental conditions. 157 genes (42 up- and 115 down-regulated) among 213 belonging to cell cycle and/or DNA replication transcripts revealed modulated activity upon at least a single type of experimental conditions (Table 1).

**Table 1:** Number of genes related to cell cycle and/or DNA replication process with expression modulated in HDFa cells by tested flavonoids or their mixtures at least in one experimental condition with respect to total number of genes key to cell cycle and/or DNA replication regulation analyzed with microarrays

Genes	Biological process		
	Cell cycle and/or replication	Cell cycle	Replication
	No. of genes		
Total (regulated and un-regulated)	213	128	112
Regulated	157	93	86
Up-regulated	42	29	17
Down-regulated	115	64	69

The fold change of gene expression ratios was ≥1.3 and ≤0.7, with a *P* value <0.05 corrected by false discovery rate, and *n* ≥ 3 were considered for flavonoid-treated cells against DMSO-treated samples

Among the tested flavonoids, genistein, kaempferol, and their mixture belonged to the treatment conditions responsible for the highest number of genes with modulated expression in fibroblasts after both 24 and 48 h (Table 2). In total, 77, 60, and 23 transcripts for 24 h and 68, 95, and 91 for 48 h handling with 100 µM genistein, 100 µM kaempferol, and mix of genistein with kaempferol (30 µM each), respectively, were affected.

**Table 2:** Number of genes altered as a function of the treatment type

	No. of genes																			
Gene expression modulation	Genistein						Kaempferol						Genistein+kaempferol		Daidzein				Genistein+daidzein	
	Concentration (μM)																			
	30		60		100		30		60		100		30 + 30		60		100		30 + 30	
	Time of exposure (h)																			
	24	48	24	48	24	48	24	48	24	48	24	48	24	48	24	48	24	48	24	48
In sum	17	29	60	50	77	68	69	82	43	91	60	95	23	91	17	84	12	55	6	65
Up-regulation	2	2	7	7	16	16	9	12	7	16	14	18	13	17	6	10	10	13	5	20
Down-regulation	15	27	53	43	61	52	60	70	36	75	46	77	10	74	11	74	2	42	1	45

Genes key to cell cycle and DNA replication regulation with expression modulated in HDFa cells upon flavonoid treatment were obtained from microarray analysis against DMSO-treated samples

Alterations in mRNA level of selected genes were considered as 0.7 ≥ FC ≥ 1.3, with a *P* value <0.05, and *n* ≥ 3

**Fig. 1:**
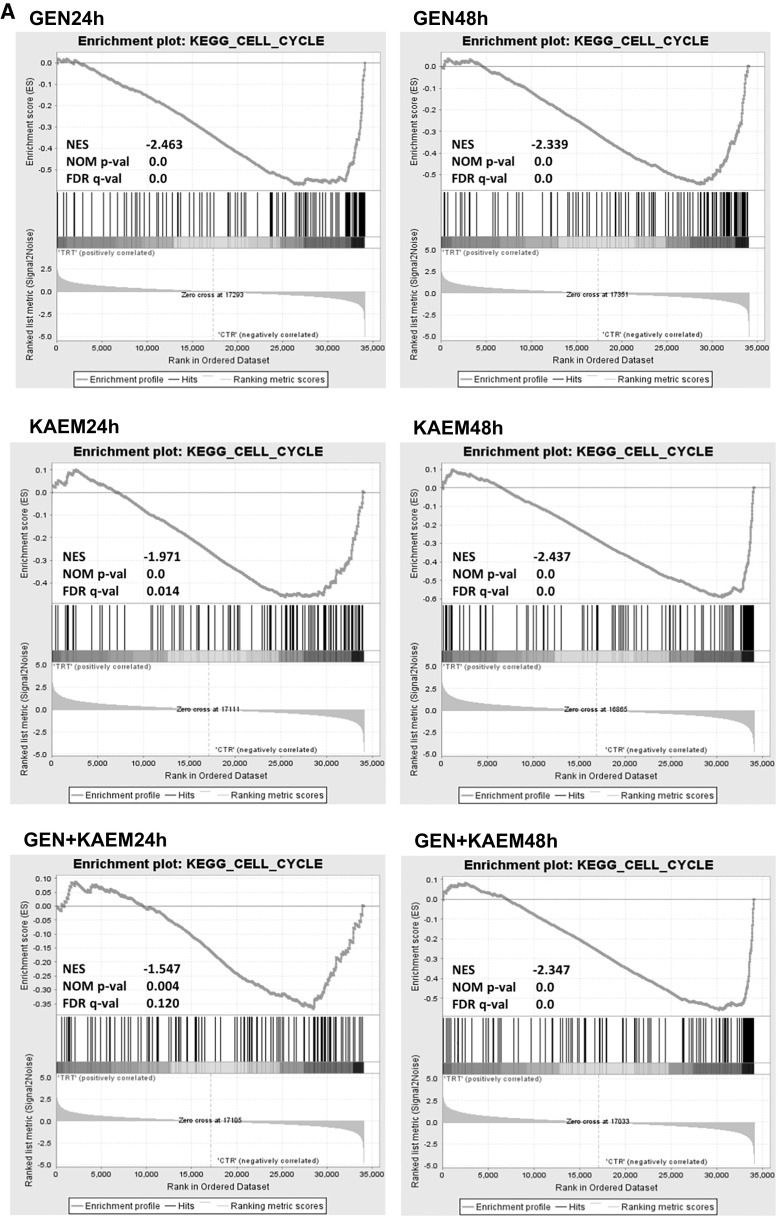

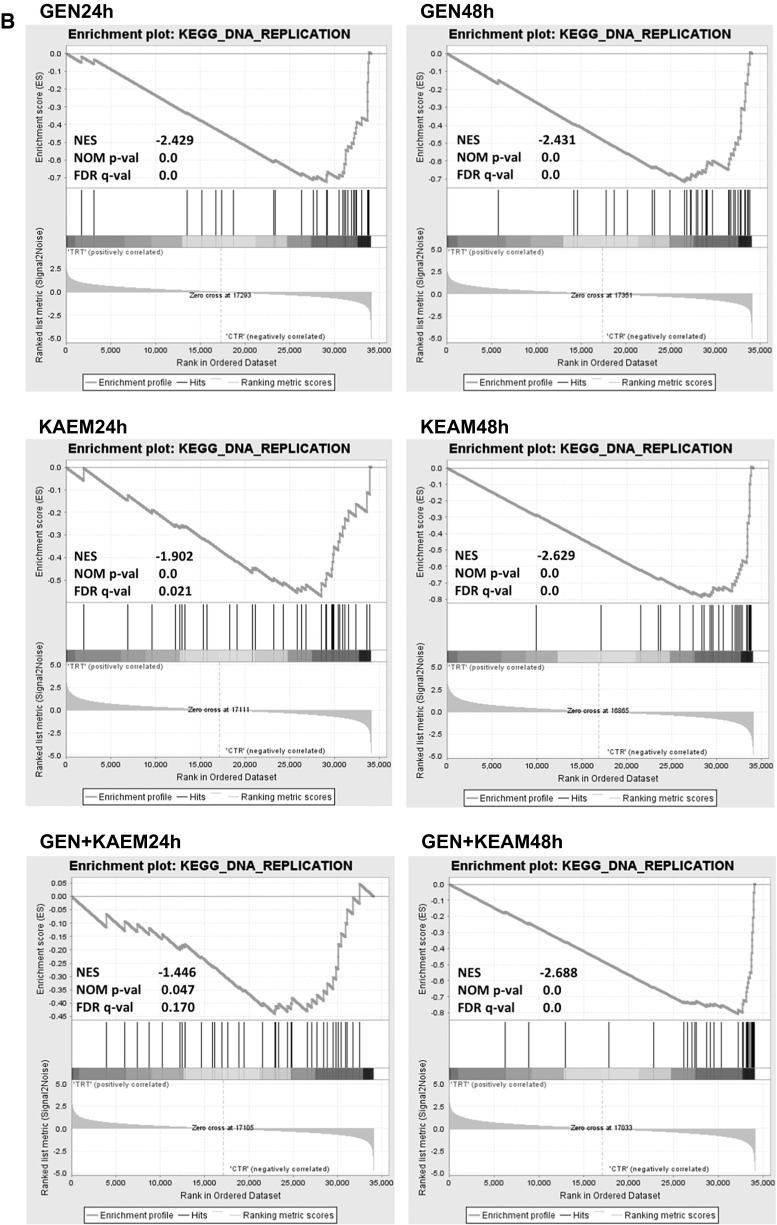
GSEA analysis of cell cycle-related (**a**) and DNA replication regulation (**b**) gene sets enriched among genes up- and down-regulated by 100 µM genistein (GEN), 100 µM kaempferol (KAEM), and mixture of them (GEN + KAEM) at 30 µM each, for 24 and 48 h in HDFa fibroblasts. Graphs show the enrichment plots generated by GSEA analysis of ranked gene expression data (*left* up-regulated, *red*; *right* down-regulated, *blue*). The enrichment score is shown as a *green line*, and the *vertical black bars* below the plot indicate the position of cell cycle or DNA replication regulation-associated genes, which are mostly grouped in the fraction of down-regulated genes. (Color figure online)

Interpretation of biological meaning of defining cell cycle- and DNA replication-associated gene sets was performed by GSEA analysis. The results showed a significant enrichment among these genes regulated by genistein, kaempferol, and genistein–kaempferol mix (Fig. 1), with normalized Enrichment Score stronger than in the case of daidzein and genistein–daidzein treatment (data not shown).

**Fig. 2:**
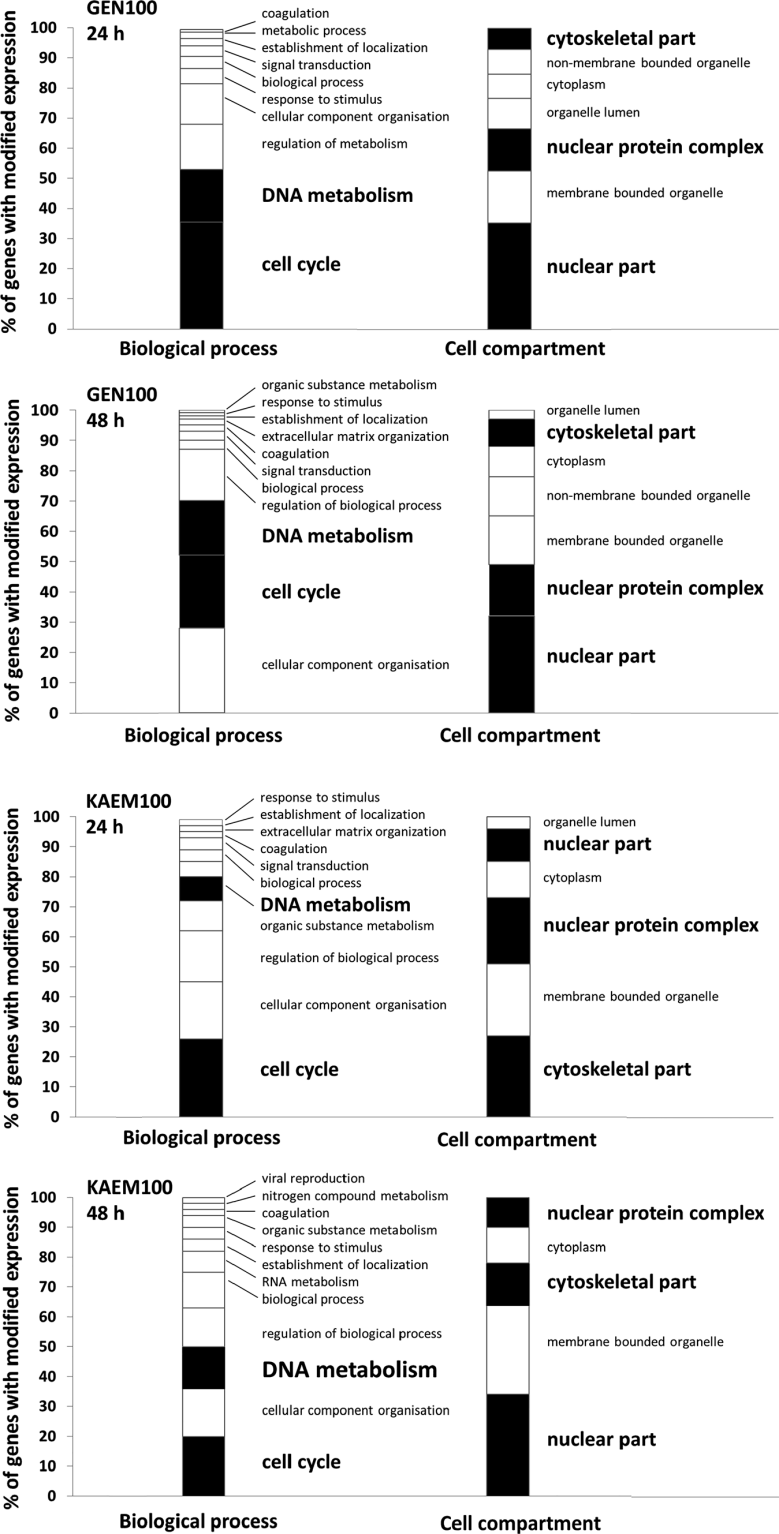

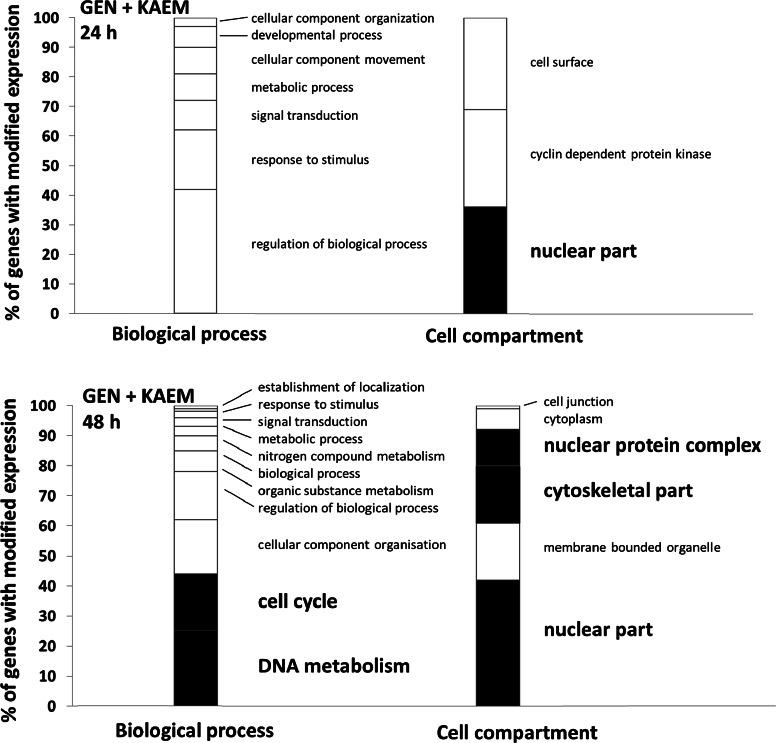
GO analysis by ‘Cellular Compartment’ and ‘Biological Processes’ category of genes with down-regulated expression upon flavonoids’ treatment (100 µM genistein, 100 µM kaempferol, and mixture of them of 30 µM each, for 24 and 48 h) of HDFa cells, with false discovery rate (FDR) <0.1, fold change ≥1.3 and below 0.7, and *P* < 0.001. All cell cycle regulation and/or DNA replication relevant cellular compartments and processes are marked in *black*, while everything else is *white*

Furthermore, the analysis of ‘Cellular Compartment’ as well as ‘Biological Processes’ terms revealed that among all tested conditions, treatment respectively with genistein, kaempferol, and genistein–kaempferol mix altered significantly the expression of cell cycle- and DNA replication-related genes (Fig. 2). These enrichments for these categories were noticeably stronger than those observed when daidzein or genistein–daidzein mix were applied (data not shown).

### Real-time qRT-PCR expression analysis of selected genes

Relative activity of 44 cell cycle and/or DNA replication-related genes, selected previously in the course of microarray analysis, in HDFa fibroblasts treated for 24 h with 100 µM genistein were examined by real-time qRT-PCR (Table 3). A custom-made human cell cycle profiler PCR array plate was utilized to assess the expression levels of particular genes and to validate microarray data. We confirmed considerable down-regulation of expression of most genes tested, with only four activated transcripts (*CDKN1A*, *CDKN1C*, *CDKN2B*, and *GADD45A*) in response to 100 µM genistein after 24 h period of its action. Among these 44 genes, either up or down-regulated, three, i.e., *MCM8*, *CDKN2A,* and *CDKN2C* gave different responses in both DNA microarray and real-time qRT-PCR analyses.

**Table 3:** Validation of microarray data of selected cell cycle- and/or DNA replication-related genes in HDFa cells by real-time qRT-PCR array

**Cell cycle genes with modulated expression (44)**	**Protein**	**FC ± SD (microarray)**	**FC ± SD (plate qRT-PCR)**
**G1 Phase and G1/S transition (3)**			
***CDC7***	***Cell division cycle 7***	***0.52 ± 0.1***	***0.28 ± 0.1***
*CDKN1A (p21)*	*Cyclin-dependent kinase inhibitor 1A*	*2.34 ± 0.2*	*1.47 ± 0.2*
*CDKN1C (p57)*	*Cyclin-dependent kinase inhibitor 1C*	*1.46 ± 0.3*	*1.30 ± 0.3*
**S Phase and DNA replication (14)**			
***CDT1***	***Chromatin licensing and DNA replication factor 1***	***0.43 ± 0.1***	***0.23 ± 0.0***
***FEN1***	***Flap structure-specific endonuclease 1***	***0.43 ± 0.1***	***0.19 ± 0.0***
***GINS2***	***GINS complex subunit 2***	***0.34 ± 0.0***	***0.20 ± 0.0***
***GINS3***	***GINS complex subunit 3***	***0.40 ± 0.1***	***0.15 ± 0.0***
***GMNN***	***Geminin, DNA replication inhibitor***	***0.62 ± 0.1***	***0.24 ± 0.0***
***MCM2***	***Minichromosome maintenance complex component 2***	***0.48 ± 0.0***	***0.20 ± 0.0***
***MCM3***	***Minichromosome maintenance complex component 3***	***0.52 ± 0.0***	***0.28 ± 0.1***
***MCM4 (CDC21)***	***Minichromosome maintenance complex component 4***	***0.43 ± 0.1***	***0.30 ± 0.0***
***MCM5 (CDC46)***	***Minichromosome maintenance complex component 5***	***0.41 ± 0.1***	***0.23 ± 0.0***
***MCM6 (Mis5)***	***Minichromosome maintenance complex component 6***	***0.61 ± 0.0***	***0.37 ± 0.0***
***MCM7 (CDC47)***	***Minichromosome maintenance complex component 7***	***0.54 ± 0.0***	***0.28 ± 0.0***
***MCM8***	***Minichromosome maintenance complex component 8***	***1.41 ± 0.2***	***0.27 ± 0.0***
***MCM10***	***Minichromosome maintenance complex component 10***	***0.40 ± 0.1***	***0.28 ± 0.0***
***POLE***	***DNA polymerase epsilon subunit 1***	***0.54 ± 0.1***	***0.33 ± 0.0***
***POLE2***	***DNA polymerase epsilon subunit 2***	***0.53 ± 0.1***	***0.32 ± 0.0***
***TOP2A***	***DNA topoisomerase 2 alpha***	***0.69 ± 0.2***	***0.13 ± 0.0***
**G2 phase and G2/M transition (6)**			
***BIRC5 (CPC)***	***Survivin, baculoviral IAP repeat-containing protein 5*** **(** ***Chromosomal Passenger Complex*** **)**	***0.39 ± 0.1***	***0.10 ± 0.0***
***CCNA1***	***Cyclin A1***	***0.78 ± 0.0***	***0.32 ± 0.2***
***CCNB1***	***Cyclin B1***	***0.42 ± 0.1***	***0.08 ± 0.0***
***CDK2***	***Cyclin-dependent kinase 2***	***0.44 ± 0.0***	***0.21 ± 0.1***
***PLK1***	***Polo-like kinase 1***	***0.38 ± 0.1***	***0.05 ± 0.0***
***TPX2***	***Microtubule-associated targeting protein for Xklp2***	***0.33 ± 0.0***	***0.09 ± 0.0***
**M Phase (3)**			
***CCNB2***	***Cyclin B2***	***0.51 ± 0.1***	***0.10 ± 0.0***
***CCNB3***	***Cyclin B3***	***0.38 ± 0.0***	***0.65 ± 0.2***
***CDC20***	***Cell division cycle 20***	***0.42 ± 0.1***	***0.08 ± 0.0***
**Cell cycle checkpoint and cell cycle arrest (14)**			
***CCNA2***	***Cyclin A2***	***0.44 ± 0.1***	***0.11 ± 0.0***
***CDC45L***	***Cell division cycle 45-like***	***0.33 ± 0.0***	***0.21 ± 0.0***
***CDK2***	***Cyclin-dependent kinase 2***	***0.44 ± 0.0***	***0.21 ± 0.1***
*CDKN1A (p21)*	*Cyclin-dependent kinase inhibitor 1A*	*2.34 ± 0.2*	*1.47 ± 0.2*
*CDKN1C (p57)*	*Cyclin-dependent kinase inhibitor 1C*	*1.46 ± 0.3*	*1.30 ± 0.3*
***CDKN2A (p16)***	***Cyclin-dependent kinase inhibitor 2A***	***2.04 ± 0.0***	***0.50 ± 0.1***
*CDKN2B (p15)*	*Cyclin-dependent kinase inhibitor 2B*	*1.34 ± 0.1*	*1.39 ± 0.1*
***CDKN2C (p18)***	***Cyclin-dependent kinase inhibitor 2C***	***1.23 ± 0.2***	***0.12 ± 0.0***
***CDKN2D (p19)***	***Cyclin-dependent kinase inhibitor 2D***	***0.70 ± 0.2***	***0.15 ± 0.0***
***CHEK1 (CHK1)***	***cell cycle checkpoint kinase 1***	***0.38 ± 0.0***	***0.26 ± 0.0***
***CHEK2 (CHK2 / RAD53)***	***Cell cycle checkpoint kinase 2***	***0.67 ± 0.1***	***0.56 ± 0.0***
*GADD45A (DDIT4)*	*Growth arrest and DNA-damage-inducible 45 (DNA-damage-inducible transcript 1)*	*1.55 ± 0.3*	*1.78 ± 0.6*
***RB1***	***Retinoblastoma 1***	***0.61 ± 0.0***	***0.40 ± 0.1***
***TP53 (p53)***	***Tumor protein 53***	***0.59 ± 0.1***	***0.74 ± 0.1***
**Regulation of the cell cycle (10)**			
***CCNA1***	***Cyclin A1***	***0.78 ± 0.0***	***0.32 ± 0.2***
***CCNA2***	***Cyclin A2***	***0.44 ± 0.1***	***0.11 ± 0.0***
***CCNB1***	***Cyclin B1***	***0.42 ± 0.1***	***0.08 ± 0.0***
***CCNB2***	***Cyclin B2***	***0.51 ± 0.1***	***0.10 ± 0.0***
***CDC20 (p55cdc)***	***Cell division cycle 20***	***0.42 ± 0.1***	***0.08 ± 0.0***
***CDC45L***	***Cell division cycle 45-like***	***0.33 ± 0.0***	***0.21 ± 0.0***
***CDK2***	***Cyclin-dependent kinase 2***	***0.44 ± 0.0***	***0.21 ± 0.1***
***E2F2***	***E2F transcription factor***	***0.30 ± 0.1***	***0.16 ± 0.0***
*GADD45A (DDIT4)*	*Growth arrest and DNA-damage-inducible 45 (DNA-damage-inducible transcript 1)*	*1.55 ± 0.3*	*1.78 ± 0.6*
***RB1***	***retinoblastoma 1***	***0.61 ± 0.0***	***0.40 ± 0.1***
**Negative regulation of the cell cycle (4)**			
***CDC7***	***Cell division cycle 7***	***0.52 ± 0.1***	***0.28 ± 0.1***
*CDKN2B (p15)*	*Cyclin-dependent kinase inhibitor 2B*	*1.34 ± 0.1*	*1.39 ± 0.1*
***CDKN2D (p19)***	***Cyclin-dependent kinase inhibitor 2D***	***0.70 ± 0.2***	***0.15 ± 0.0***
***TP53 (p53)***	***tumor protein 53***	***0.59 ± 0.1***	***0.74 ± 0.1***
**Chromosome segregation (3)**			
***BIRC5 (CPC)***	***Survivin, baculoviral IAP repeat-containing protein 5 (Chromosomal Passenger Complex)***	***0.39 ± 0.1***	***0.10 ± 0.0***
***CENPA***	***Centromere protein A***	***0.37 ± 0.1***	***0.06 ± 0.0***
***INCENP***	***Inner centromere protein antigens***	***0.67 ± 0.1***	***0.15 ± 0.0***
**Mitotic checkpoint regulators/spindle assembly checkpoint (SAC) (2)**			
***BUB1***	***Mitotic checkpoint serine***	***0.26 ± 0.1***	***0.10 ± 0.0***
***TTK***	***TTK protein kinase***	***0.46 ± 0.1***	***0.10 ± 0.0***

The microarray and real-time qRT-PCR array data represent fold change (FC, greater or equal to 1.3, and below 0.7, with *P* <0.001) averaged values ±SD from *n* ≥ 3 and denote significant differences for samples treated with 100 μM genistein against non-treated samples for 24 h, with respect to reference gene *GAPDH* of constant expression level

The symbols of genes that were down-regulated are bolded in italic and up-regulated normal in italic

### Cytotoxicity of genistein and its effect on migration and growth of fibroblasts and cell cycle progression

**Fig. 3:**
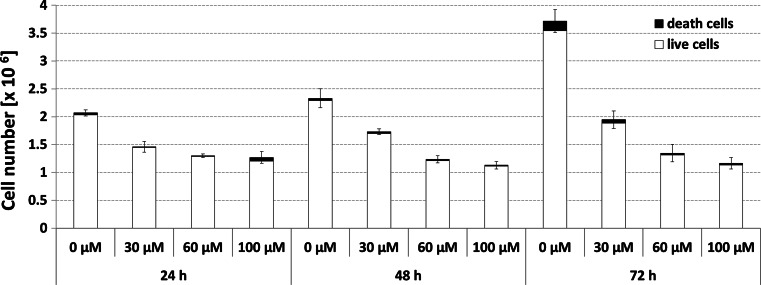
Determination of count and viability of HDFa cells treated with 30, 60, and 100 μM genistein for 24, 48, and 72 h. Data are represented as mean, and *bars* show SD values of experiments run in triplicate. Cell count test gave statistical differences among experimental groups using one-way ANOVA followed by Tukey’s HSD Post-Hoc (*P* < 0.00001), while for cell viability, no significant alterations were detected except for cells treated for 72 h with 60 and 100 μM genistein versus control with significance value of *P* < 0.05 as determined by one-way ANOVA

**Fig. 4:**
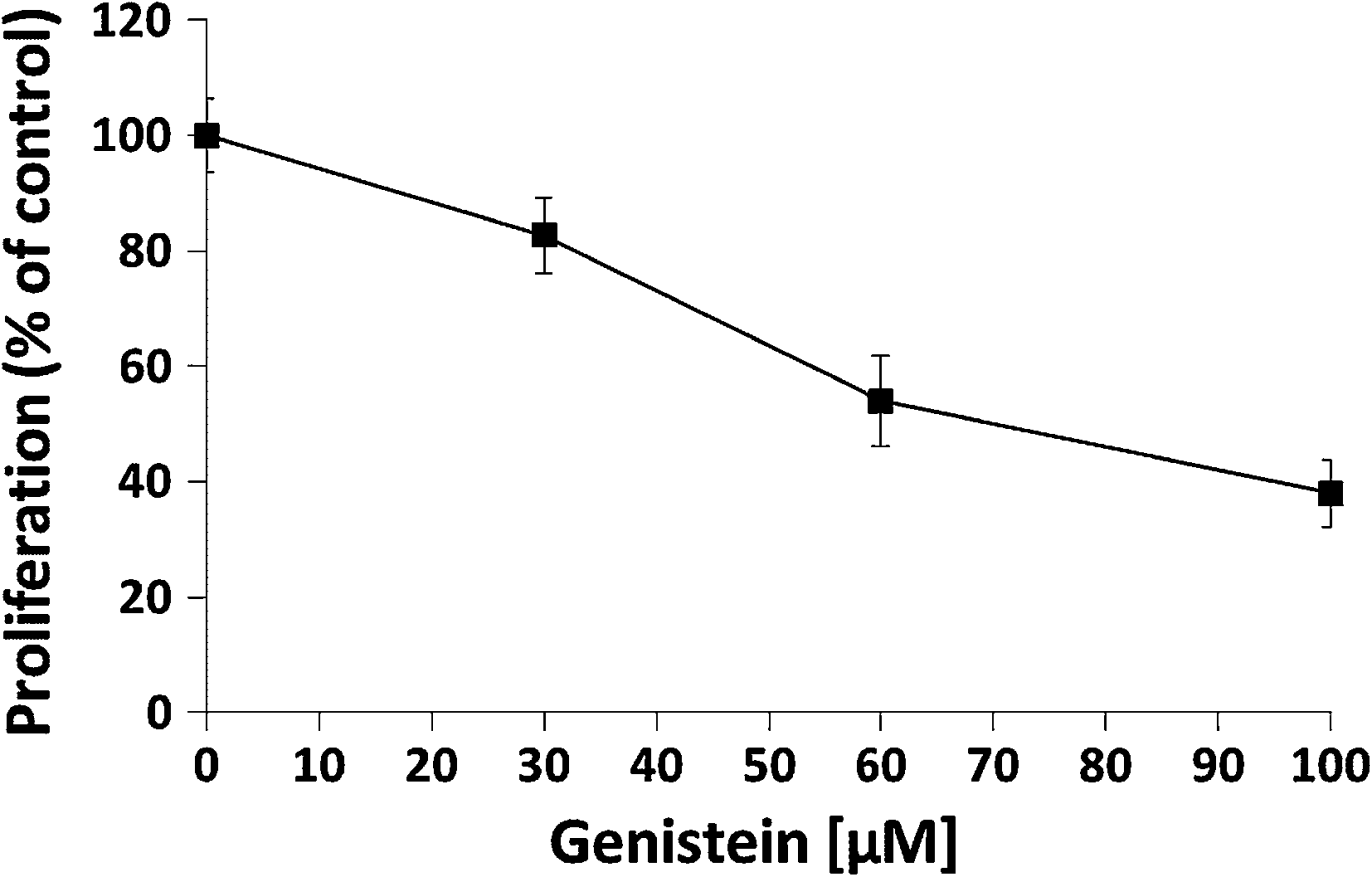
Effect of genistein on growth of HDFa fibroblasts determined for cultures treated for 7 days with the tested compound of 30, 60, and 100 μM, and calculated as ratio, comparing to the DMSO-treated control. *Data* are presented as mean values ±SD from at least triplicate wells and three independent experiments

To investigate the cytotoxicity effect of genistein, we measured metabolic activity of HDFa cells treated with different concentrations (30, 60, and 100 μM) of genistein for different periods of time (24, 48, and 72 h). The cytotoxicity assessed as LC25, 50, and 75 is described in Table 4. The lethal concentration values were >100 μM, except for LC25 values of 59 and 41 μM genistein after 48- and 72-h incubation periods, respectively. The viability of fibroblasts was additionally assessed with the use of automated cell analyzer system. As seen in Fig. 3, the cell viability did not change remarkably at the tested conditions, although the total cell number was reduced. Moreover, the growth inhibitory effect of genistein was studied in HDFa cells treated with various concentrations (30, 60, and 100 μM) of compound for 7 days (Fig. 4). It should be noted that the anti-proliferative activity of genistein is concentration dependent.

**Table 4:** Cytotoxic activity of genistein expressed as LC25, 50, or 75 index value, i.e., concentration of the tested compound (μM) that is lethal to 25, 50, or 75 % of cells in a culture exposed to genistein for 24, 48, and 72 h

Time of exposure (h)	LC_25_ (μM)	LC_50_ (μM)	LC_75_ (μM)
24	>100	>100	>100
48	59	>100	>100
72	41	91	>100

In addition, the analysis of genistein effect on fibroblast migration by in vitro scratch assay showed acceleration of movement of cells treated with this isoflavone when coupled with control untreated fibroblasts, with time- and dose-dependent alterations (Table 5), thus revealing in vitro wound healing properties of genistein.

**Table 5:** The effect of genistein on the migration of fibroblasts in a culture supplemented with 30, 60, and 100 μM of genistein or 0.05 % DMSO as control

Genistein (μM)	Fibroblast migration (% of distance travelled by control cells)				
	0 h	6 h	12 h	18 h	24 h
30	121 ± 13	96 ± 31	100 ± 37	99 ± 41	113 ± 53
60	111 ± 15	107 ± 28	114 ± 36	102 ± 40	115 ± 53
100	110 ± 16	106 ± 23	115 ± 31	120 ± 35	143 ± 65

The rate of cell migration was determined for the cells by measuring the distance traveled during the desired time frame of incubation, i.e., at 0, 6, 12, 18, and 24 h

The data represent averaged values ±SD of experiments run in triplicate from at least 100 measurements and are denoted as % of control

Statistical differences among all experimental groups using one-way ANOVA followed by Tukey’s HSD Post-Hoc (*P* < 0.00001) were observed

**Fig. 5:**
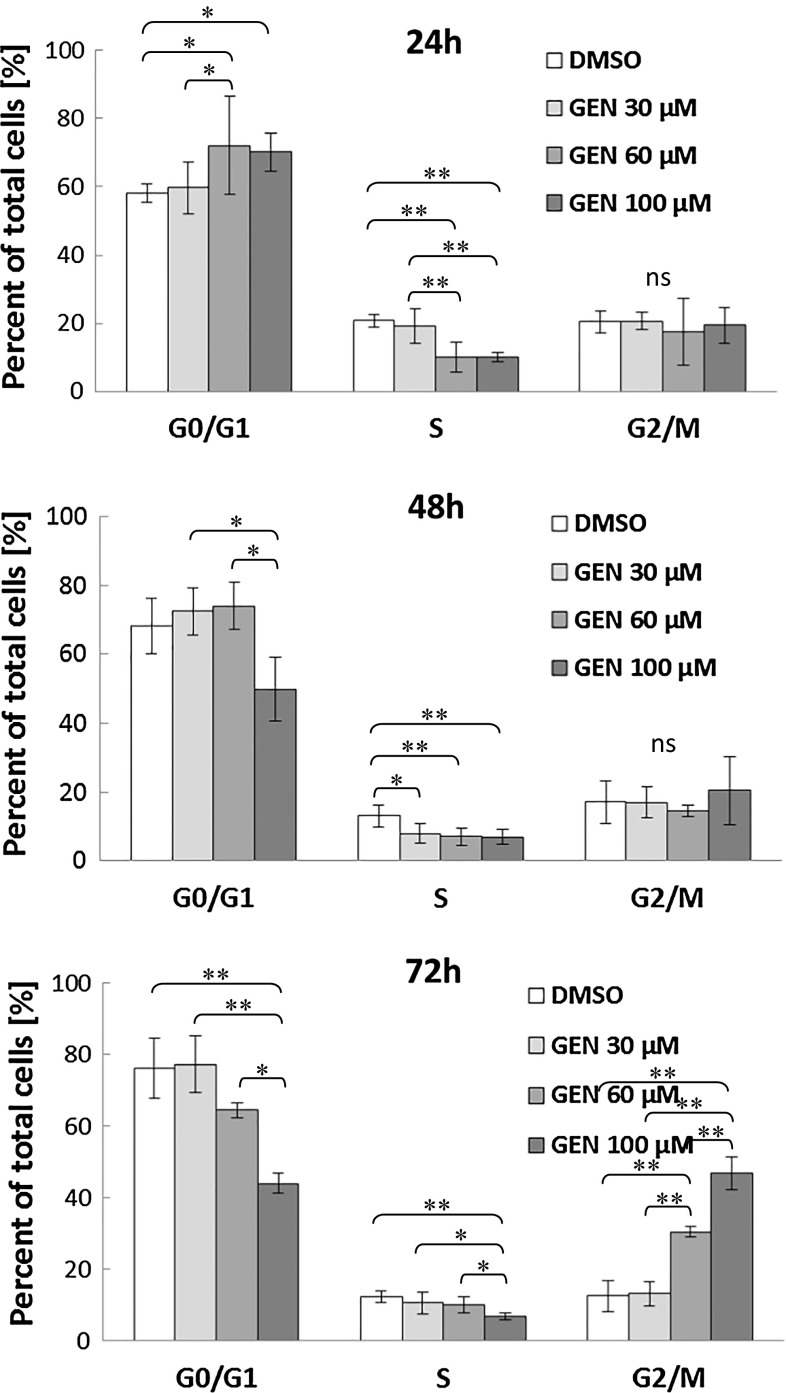
Effect of genistein on cell cycle progression in HDFa fibroblasts. After 24, 48, and 72 h from the addition of 30, 60, or 100 μM genistein, the percentage of G0/G1, S, and G2/M was calculated. Data are presented as a ratio of total cells counted and represent mean values ±SD of at least triplicate experiments. Comparisons among groups were performed using a one-way ANOVA with Tukey HSD Post-Hoc (*P ≤ 0.05 and **P ≤ 0.001). Ns stands for not significant

Besides, the effect of genistein on cell cycle progression of HDFa fibroblasts after 24, 48, and 72 h following the addition of this isoflavone was examined. As shown in Fig. 5, in fibroblasts treated with 60 and 100 μM genistein, the percentage of G0/G1 cells initially (at 24 h) increased in respect to DMSO and 30 μM genistein and thereafter gradually decreased after 48 and 72 h; on the contrary, there was induction of untreated cells. As to the S phase, genistein slightly decreased the population of HDFa fibroblasts in a dose-dependent manner, while it induced cell number in the G2/M phase, especially at 72 h. The concentrations greater than 30 μM genistein were necessary to alter cell cycle progression, with maximal accumulation of G2/M cells observed at 100 μM. However, the increasing percentage of cells in G2/M stage in response to growing concentration of genistein at 72 h may also result from anti-proliferative effect of the tested compound (Table 4).

## Discussion

Selected flavonoids were used in cell culture studies to examine their effect on cell cycle and DNA replication. Plant metabolites are compounds met by human cells as humans usually take them with food. Moreover, some of these compounds are either used or proposed for the use in various therapeutic approaches, since they may significantly influence physiology of humans. This is plausible as many plant metabolites have various biological activities, which are, however, rather poorly understood at the molecular level. Therefore, we aimed to investigate mechanisms of influence of these substances on cell division cycle and DNA replication in exemplar eukaryotic system–normal human fibroblasts. We have chosen flavonoids as results of previous studies indicating that they can significantly influence DNA synthesis (see below). The idea to use fibroblasts from a healthy individual arose from the fact that vast majority of studies on effects of flavonoids are performed on cancer cells. What is more, the knowledge regarding the influence of flavonoids on human cell cycle and DNA replication in normal cells, especially in non-neoplastic fibroblasts, is desired as these compounds have extensive medical significance and are used for treatment of some common diseases, due to their proven ability to inhibit specific enzymes, to simulate some hormones and neurotransmitters, and to scavenge free radicals.

In our study, genistein gave the most spectacular results. It was suggested previously that inhibition of DNA replication by this compound may be due to inactivation of DNA topoisomerases ([3] and references therein). Moreover, genistein was demonstrated to induce the arrest of the cell cycle in cancer cells [11]. We performed systematic studies to recognize pathways leading to the final effect of cell cycle and DNA replication impairment by genistein and other flavonoids. The transcriptome of the cell line HDFa was profiled with Illumina’s Human HT-12 v3 Expression BeadChips. Since genistein gave the most interesting results, this isoflavone was studied in more detail.

We confirmed that genistein influences cell cycle control in the human fibroblast model. More importantly, the results from DNA microarrays were confirmed by an independent, quantitative method, the real-time Reverse Transcription PCR. Both microarray and real-time qRT-PCR analyses indicated that genistein significantly influences expression of many genes involved in cell cycle control at its various stages and DNA replication regulation (Table 3).

Genistein is known to inhibit proliferation of human cancer cells [13], which made it a potential anti-cancer therapeutic. Importantly, at concentrations low enough to influence cancer cells, there is only a minor effect on normal cells. As it has attracted attention because of its beneficial effects on prevention of metabolic disorders related to cardiovascular disease (CVD), obesity, and also diabetes, the side effects and warnings of genistein have ever been considered. Some results could suggest that genistein might impair immunity by its negative effects on mouse thymus gland and various white blood cells [24]. Moreover, in vitro studies have shown genistein to induce apoptosis of testicular cells at certain levels, thus raising concerns about effects it could have on male fertility [25]. However, a recent work indicated that isoflavones had no observable effect in healthy males given isoflavone supplements daily over a 2-month period [26]. Similarly, no significant adverse effects of genistein were observable in studies on two other groups, even when treatment with this isoflavone was as long as 6 or 12 months [27, 28].

The hypothesis on halting of DNA replication through genistein-mediated inhibition of topoisomerase activity, presented for the bacterial model, was also extended to eukaryotic cells [17]. However, our transcriptomic studies indicated that in human cells cultured in the presence of genistein, no alterations in activity of all four genes, *TOPA1*, *TOPA2*, *TOPB1,* and *TOPB2*, encoding for DNA topoisomerase were observed. On the contrary, the efficiency of expression of genes coding for MCM2-7 and MCM10 helicases, as well as some other proteins involved in the S phase control, was significantly reduced (Table 3). This may indicate that effects of genistein on DNA replication are not only restricted to its action on topoisomerases, but also include down-regulation of expression of genes required for DNA synthesis. Furthermore, levels of mRNA of genes coding for proteins involved in chromosome segregation were also less abundant in the presence of genistein.

Interestingly, we found that genistein modulates (in most cases down-regulates, but in some up-regulates) expression of a relatively large group of genes, whose products are involved in different phases of the cell cycle (the G1 phase and G1/S phase transition, G2 phase, and G2/M phase transition, M phase), as well as in regulatory processes, like cell cycle checkpoint and cell cycle arrest, mitotic checkpoint, and spindle assembly checkpoint. Some recent reports also indicated that genistein influences expression of such genes. For example, up-regulation of expression of *p21* (*CDKN1A*) in prostate cancer cells has been described previously [29], and similar results are shown here in normal human fibroblasts. Increased expression of *BDNF* was detected in genistein-treated hippocampus neuronal cells [30], and decreased levels of the miR-223, a hematopoietic-specific microRNA with crucial function in myeloid lineage development, promoting granulocytic and suppressing erythrocytic differentiation, were found in pancreatic cancer cells in response to this isoflavone [31]. Moreover, in accordance to results presented in this study, genistein was found to induce G2/M cell cycle arrest and apoptosis of human ovarian cancer cells via activation of DNA damage checkpoint pathways [8].

Genistein is known as an inhibitor of various kinases, such as serine, threonine, tyrosine, histidine protein kinases, and the HER-2 kinase [32]. This isoflavone has been shown to inhibit the activation of NF-κB and Akt signaling pathways, both of which are important for cell survival via maintaining a homeostatic balance between cell survival and apoptosis. Therefore, it is likely that modulation of expression of genes shown in Table 3 is due to genistein-mediated inhibition of particular signal transduction pathways.

In conclusion, our results of transcriptomic analysis supported by real-time qRT-PCR, obtained with the human fibroblasts model, together with results of other researchers who focused on particular genes in other cells, indicate that genistein may regulate cell cycle and DNA replication in human cells due to modulation of expression of a relatively large group of genes whose products are involved in these processes. This regulation may also be accompanied by direct action of genistein on important proteins, like topoisomerases or cyclins; however, this assumption requires further verification.
